# Peak Occurrence of Retinal Detachment following Cataract Surgery: A Systematic Review and Pooled Analysis with Internal Validation

**DOI:** 10.1155/2018/9206418

**Published:** 2018-11-22

**Authors:** Rabea Kassem, Yoel Greenwald, Asaf Achiron, Idan Hecht, Vitaly Man, Liron Ben haim, Amir Bukelman

**Affiliations:** ^1^Department of Ophthalmology, Kaplan Medical Center, Rehovot and the Hebrew University of Jerusalem-Hadassah Medical School, Jerusalem, Israel; ^2^Department of Ophthalmology, Edith Wolfson Medical Center, Holon, Israel; ^3^Sackler School of Medicine, Tel Aviv University, Tel Aviv, Israel; ^4^Department of Ophthalmology, Soroka University Medical Center, Ben-Gurion University of the Negev, Beer-Sheva, Israel

## Abstract

**Introduction:**

Timing of retinal detachment (RD) following cataract surgery is of importance for both diagnostic and prognostic factors. However, results on RD onset-time following cataract surgery have been conflicting.

**Method:**

A systematic pooled analysis of the literature regarding timing of retinal detachment following cataract surgery. Outcomes were verified against an independent dataset.

**Results:**

Twenty-one studies, reporting on rates of RD in 3,352,094 eyes of 2,458,561 patients, met our inclusion criteria and were included in the analysis. The mean pooled time to RD following surgery was 23.12 months (95% CI: 17.79–28.45 months) with high heterogeneity between studies (*I* ^2^=100%, *P* < 0.00001). Meta-analytic pooling for the risk of retinal detachment revealed a risk of 1.167% (95% CI: 0.900 to 1.468, *I* ^2^=99.50%, *P* < 0.0001). A retrospective chart review identified 54 pseudophakic RD cases (mean age 65.5, 59.3% males). The 95% confidence interval for the mean time to RD was 3.1–6.75 years.

**Conclusions:**

The interval between cataract surgery and RD in a pooled analysis revealed a mean time of approximately 1.5–2.3 years. However, there was high variability between studies. Validation based on our local results showed similar yet slightly longer time frames. Timing of pseudophakic retinal detachment might direct appropriate follow-up, assisting in earlier detection.

## 1. Introduction

The risk of retinal detachment (RD) following cataract surgery has been estimated at 0.7%, much higher than the rate of 0.08% for rhegmatogenous RD in the general population [1]. This higher risk of RD following cataract surgery has important public health implications as the absolute number of cataract surgeries performed is steadily increasing worldwide [2].

The timing of RD following cataract surgery is of importance for both diagnostic and prognostic factors. However, results on the RD onset-time following cataract surgery have been conflicting. For example, a study from France analyzing over 2.5 million cases showed that the risk of RD increased in a nearly linear manner over time following surgery [3]. On the other hand, a 2012 study from England of 62,298 cases demonstrated that most RD cases occurred within the first 2 postoperative years [4]. To our knowledge, no systematic analysis has been published regarding the time interval between cataract surgery and RD. Therefore, the aim of this study is to perform a systemic literature review combined with pooled analysis on the temporal occurrence of RD after cataract surgery and verify the results against an independent dataset.

## 2. Methods

### 2.1. Systematic Review

#### 2.1.1. Literature Searches Methods

A systematic search was conducted using Cochrane Library and MEDLINE, PubMed, ClinicalTrials.gov, metaRegister of Controlled Trials (http://www.controlled-trials.com), WHO International Clinical Trials Registry Platform (http://www.who.int/ictrp/search/en), and Google Scholar with the following keywords: Cataract, OR cataract surgery, OR cataract extraction, OR phacoemulsification, AND Retinal Detachment, OR, RD, and OR detachment.

#### 2.1.2. Eligibility Criteria

The aim of this review was to identify studies which relate to effects of cataract removal on RD onset. We included studies meeting the following criteria: (1) studies examining temporal occurrence of RD after cataract surgery; (2). studies using modern method of phacoemulsification techniques; (3) written in English; (4) full publications (not an abstract or letter to the editor). Our exclusion criteria included (1) case reports and nonempirical opinion articles; (2) clear\refractive lens exchange; and (3) pediatric cases.

#### 2.1.3. Screening and Synthesis

The review process was conducted under the guidance of the PRISMA (Preferred Reporting Items for Systematic Reviews and Meta-Analyses) criteria to support reporting [5]. Two reviewers (RK and VM) independently conducted the search for relevant publications. Selected publications were then approved by a senior investigator (AA). Individual studies were graded using the Scottish Intercollegiate Guideline Network (SIGN) assessment system for individual studies as implemented for Preferred Practice Patterns by the American Academy of Ophthalmology. Supplemental Figure 1 shows the flow diagram of the inclusion process [6].

#### 2.1.4. Statistical Analysis

Meta-analyses were performed using the Cochrane Collaboration Review Manager Software version 5.3.5. We pooled the study-specific outcome estimates and their standard errors in random-effects pooled-analyses. We assessed heterogeneity with *I* ^2^ and Cochran's Q with corresponding *P* values, and values less than 0.10 were considered significant for heterogeneity. When the *I* ^2^ estimate was equal to 25%, 50%, and 75%, we interpreted as indicating the presence of low, moderate, and high heterogeneity, respectively. For assessing continuous outcomes such as the time interval between CS and RD, the generic inverse variance method was used. Pooled analysis was used in order to avoid problems arising from simple pooling [7]. Graphs were created using Medcalc software version 16 (Mariakerke, Belgium). Unless otherwise specified, data are presented as mean ± standard deviation.

### 2.2. Retrospective Validation Analysis

As a way to independently verify the accuracy of our methods and insure that no errors were introduced during the review, extraction, or analysis processes, we compared the outcomes against separate, independent dataset. This dataset was based on patients older than 40 years of age who underwent RD surgery between January 2013 and August 2014 at the Kaplan Medical Center in Israel. We included only patients with a follow-up of at least 6 months. Routinely collected medical data included the principal diagnosis, secondary diagnoses, and procedures performed. We extracted sociodemographic variables, including age and gender. Eye characteristics, including high myopia and history of eye trauma, were collected. T-tests were conducted for continuous variables and chi-squared for categorical variables. Bivariate correlation was calculated for continuous variables (Pearson correlation). Logistic regression analysis was conducted to predict the onset of RD at 1, 3, 5, and 9 years. *P* values less than 0.05 were considered statistically significant. The research followed the tenets of the Declaration of Helsinki, and approval was obtained from the Kaplan Medical Center Ethics Committee.

## 3. Results

Following the systematic review, 21 publications met our inclusion criteria which were published from 1997 to 2017 (Supplemental Table 1). A flow diagram of the inclusion process is available in Supplemental Figure 1.

### 3.1. Time to the Development of Retinal Detachment

**Figure 1:**
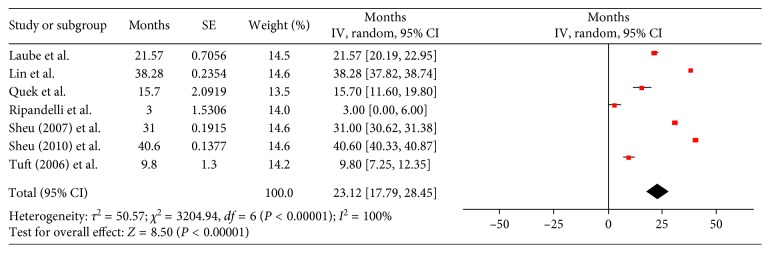
Forest plot for the time to retinal detachment. Size of the squares is proportional to the number of cases in the study. Error bars represent 95% confidence interval (95% CI). The diamond shape represents the pooled estimate. The mean pooled time from surgery to RD was 23.12 months (1.9 years, 95% CI: 17.79–28.45 months) with high heterogeneity between studies (*I* ^2^=100%, *P* < 0.00001, Tau^2^ = 50.57).

Twenty-one studies reported on the time to RD, with high variability in the reported times. Nineteen studies reported a mean value which ranged from 1.46 to 109 months; however, most (12/19) reported on a mean time which was between 12 and 40 months (1 to 3.3 years). The largest study by Daien et al. reported an interquartile range of 2.5 months to 2 years [3]. Peak occurrence was mostly reported following a few months to years; however, some studies reported that the risk remains high relative to a phakic population even at follow-up periods of over a decade. Only 7 studies reported variance metrics (standard deviations and confidence intervals) when reporting the time to the development of RD, enabling meta-analytic pooling [8–14]. The mean pooled time from surgery to RD was 23.12 months (1.9 years, 95% CI: 17.79–28.45 months) with high heterogeneity between studies (*I* ^2^=100%, *P* < 0.00001, Tau^2^ = 50.57). These results are illustrated in Figure 1.

### 3.2. Risk of Retinal Detachment

**Figure 2:**
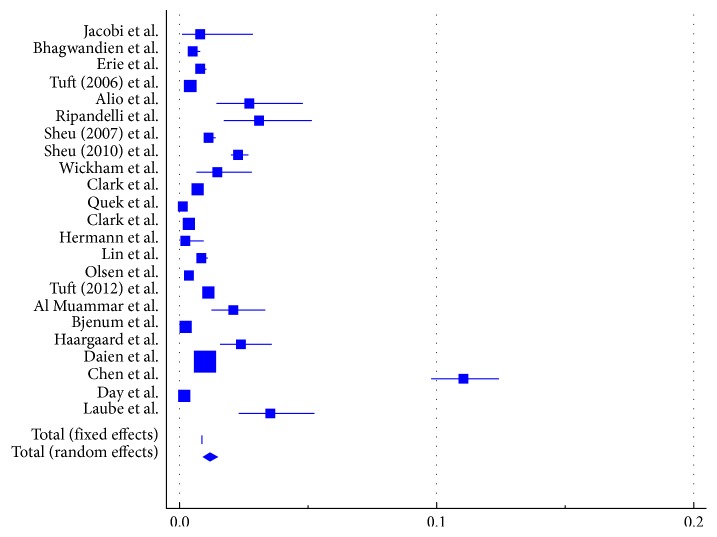
Forest plot for the risk of retinal detachment. Squares represent the proportion of patients who developed retinal detachment following cataract surgery at last available follow-up. Size of the squares is proportional to the number of cases in the study. Error bars represent 95% confidence interval (95% CI). The diamond shape represents the pooled estimate. The random-effect pooled estimate was 1.167% (95% CI: 0.900 to 1.468). Heterogeneity was significant (*I* ^2^=99.50%, *P* < 0.0001, 95% CI for *I* ^2^ = 99.43 to 99.55).

Retinal detachment rates were reported in 23 publications that met our inclusion criteria (Supplemental Figure 1 and Supplemental Table 1) with a total of 3,352,094 eyes of 2,458,561 patients [3, 4, 8–26]. However, most eyes were reported by a single study by Daien et al. which reported on 1,787,021 individual cases. The weighted mean of the total follow-up period was 45 months (3.8 years, range: 3 months to 10 years, *I* ^2^=96%). Meta-analytic pooling for the risk of retinal detachment revealed a risk of 1.167% (95% confidence interval (CI): 0.900 to 1.468, *I* ^2^=99.50%, *P* < 0.0001, 95% CI for *I* ^2^ = 99.43 to 99.55) as illustrated in Figure 2.

To test for the specificity of our results, we repeated the analyses excluding the large study by Daien et al. [3]. Results remained similar to a risk of retinal detachment of 1.183% (95% CI: 0.898 to 1.507, *I* ^2^=99.07%, *P* < 0.0001, 95% CI for *I* ^2^ = 98.91 to 99.20).

Several studies reported on the age of patients (Supplemental Table 1). However, only three studies reported on the individual ages in the group which developed RD compared with those who did not. In all three studies, it appears that as age increases, the time to RD becomes shorter (illustrated in Supplemental Figure 2). However, these represent a small sample size, too small to enable statistical analyses.

### 3.3. Validation Analysis

Out of 139 RD cases, we included in the analyses 54 cases that were pseudophakic. We had available data on 34 patients regarding the time interval to RD. Clinical characteristics of the study population is summarized in Table 1. Of all pseudophakic RDs analyzed in this study, 25% occurred within 0.7 years after surgery, 50% within 3.1 years, 75% within 6.8 years, and 90% within 14 years. The 95% confidence interval for the mean time interval between cataract surgery and RD was 3.1–6.75 years. No relations were found between age (*r*=−0.03, *P*=0.86, Pearson correlation), gender (*P*=0.30, T-test) or retinal surgery anatomical success (*p*=0.75, T-test), and the interval time from cataract surgery to RD. Logistic regression analysis failed to predict timing of RD based on clinical parameters (age, gender, and complicated cataract surgery).

## 4. Discussion

In this study, we used a systematic review combined with pooled analysis to assess the mean time interval after cataract surgery during which RD typically occurs. The pooled analysis revealed a mean time of approximately 1.5–2.3 years. Our retrospective validation analysis showed slightly longer time frames of about 4 years.

Cataract surgery is an independent risk factor for RD due to postsurgical anatomical and biochemical alterations in the vitreous. Anatomically, following the removal of the native lens, there are changes in the vitreous volume possibly affecting its mobility [24]. In addition, critical biochemical changes including differences in proteome, viscosity, and macromolecules in the vitreous humour were found and may lead to a posterior vitreous detachment (PVD), a known risk factor for RD [27].

Most studies in our review report decreasing rates of RD over time with a peak at a few months or years. Our results appear to support this notion considering the large range of interval periods from cataract surgery to RD (0.5–20 years with a mean of 4.9 years). Our local results indicate that a long follow-up is needed, as we did not find any clinical parameter to predict RD timing. This supports the reports by Hermann et al. who claimed that RD following cataract surgery is well underestimated and that follow-up of 8 years would include only 84.5% of all pseudophakic RD cases [24].

However, such a long follow-up may be difficult to adhere to. Potamitis et al. found that the frequency of nonattendance at outpatient ophthalmology clinics is about 10% of appointments and that roughly 18% of these were due to inattention to the date of the scheduled meeting [28]. Timing of pseudophakic RD onset is important for diagnostic and prognostic factors. It can direct precise period guidelines for follow-up or for scheduled reaching-out to patients regarding RD's signs and symptoms. In addition, locating the time period where patients are at the highest risk for RD may assist in detecting retinal break which may lead to earlier intervention. As technology emerges, the use of automatic alerts and reminders to notify clinicians and patients about appointments, in the form of text messages or emails, might improve attendance rates for appointments [29].

To note, all of the articles discussed in this study display the same trend—there is an increased risk of RD after cataract surgery in comparison to normal population; however, heterogeneity is seen between the studies in our analysis regarding the mean time interval between cataract surgery and RD and the exact cumulative risk for RD. This might be due to different populations (including high risk populations) of patients as well as different surgical techniques, the year during which the procedure was performed, and the mean period of follow-up. When assessing high risk populations separately (high myopia and intraoperative complications), RD occurred earlier. Day et al. reported that pseudophakic RD occurred on average 44 days following posterior capsular rupture [22]. In addition, Alio et al. reported that almost 1% and 2% of high myopic patients will exhibit RD 6 months and 12 months following cataract surgery with posterior capsular tear, respectively [16]. Furthermore, the risk for RD tends to be higher among younger patients, with the risk reaching 3.64% at 4 years after cataract surgery among patients 40–54 years old, as reported by Daien et al. [3] Laube et al. reported an overall cumulative incidence of 3.55% among patients younger than 61 years old, at a mean duration of 3.6 years from surgery.

This study has several limitations. First, it includes many studies that differ from each other by the year of publication, the type of population included, and the mean time of follow-up. The range of timing to RD reflects this heterogeneity. Second, one of the studies we included contains a significantly larger population. In order to deal with that limitation, we analyzed the data twice, with this study and without it. Third, publications may be intrinsically biased to report success rather than failure, a phenomenon known as publication bias; therefore, the risks of developing RD after cataract surgery should be considered underestimated in this review.

To conclude, the time interval between cataract surgery and RD in a pooled analysis revealed a mean time of approximately 1.5–2.3 years. There was a high variability between the studies; however, most of them reported a mean time that ranged from 12 to 40 months. Validation based on our local results showed similar yet slightly longer time frames. Timing of pseudophakic retinal detachment might direct appropriate follow-up, assisting in earlier detection.

## Figures and Tables

**Table 1:** Baseline characteristics and surgical procedures performed.

Variable	All patients (*n*=54)
Mean age (years)	65.5
** **≥50	88.9%
** **<50	11.1%
Gender	
** **Male	59.3%
** **Female	40.7%
Symptoms duration	
** **≤1 weeks	58.9%
** **>1 weeks	41.1%
Trauma	11.1%
Complicated cataract surgery	25.9%
Retinal characteristics	
** **Macula	
** **On	38.5%
** **Off	61.5%
** **Inferior tears	18.5%
** **Lattice degeneration	7.4%
** **PVR	11.1%
Surgical procedure	
** **PR	35.2%
** **PPV	63%
** **SB	1.8%

Abbreviations: PVR, proliferative viteroretinopathy; PR, pneumatic retinopexy; PPV, pars plana vitrectomy; SB, scleral buckling.

## Data Availability

The pooled analysis data used to support the findings of this study are included within the supplementary information file. The retrospective data used to support the findings of this study are available from the corresponding author upon request.

## Supplementary Materials

Supplementary Materials Supplemental Table 1: studies included in the analysis. Supplemental Figure 1: flow diagram of the inclusion process. Supplemental Figure 2: relationship between age of patients which developed RD and time to RD.
