# Supplementary material for: Peak Occurrence of Retinal Detachment following Cataract Surgery: A Systematic Review and Pooled Analysis with Internal Validation
Source: J Ophthalmol. 2018 Nov 22;2018:9206418. doi: 10.1155/2018/9206418 (PMC6282121; doi:10.1155/2018/9206418)
Supplement: Supplementary Materials — Supplemental Table 1: studies included in the analysis. Supplemental Figure 1: flow diagram of the inclusion process. Supplemental Figure 2: relationship between age of patients which developed RD and time to RD. [file 9206418.f1.zip › 9206418.f1/Supplemental Figure 1 I.H_6.7_JOPH_2525913.docx]

Supplemental Figure 1. Flow Diagram of the Inclusion Process

Records identified through database searching
(n =58 )

## Screening

## Included

## Eligibility

## Identification

Additional records identified through other sources
(n = 0)

Records after duplicates removed
(n = 46)

Records screened
(n = 28)

Records excluded as irrelevant to our subject
(n = 7)

Articles assessed for eligibility
(n = 21)

Articles included in pooled-analysis
(RD incidence n = 20, Onset of RD following cataract surgery n=7)

Full-text articles excluded
(n = 1)
