# Supplementary material for: Peak Occurrence of Retinal Detachment following Cataract Surgery: A Systematic Review and Pooled Analysis with Internal Validation
Source: J Ophthalmol. 2018 Nov 22;2018:9206418. doi: 10.1155/2018/9206418 (PMC6282121; doi:10.1155/2018/9206418)
Supplement: Supplementary Materials — Supplemental Table 1: studies included in the analysis. Supplemental Figure 1: flow diagram of the inclusion process. Supplemental Figure 2: relationship between age of patients which developed RD and time to RD. [file 9206418.f1.zip › 9206418.f1/Supplemental Table 1_JOPH_2525912.docx]

Supplemental Table 1. Studies Included in the analysis

| No | Author | year published | number of patients  (number of eyes) | mean follow up period (months) | cumulative risk for RD | mean interval between CS and RD | population | type of surgery |
| --- | --- | --- | --- | --- | --- | --- | --- | --- |
| 1 | Jacobi et al.[[27](#_ENREF_27)] | 1997 | 190 (253) | 45.6 ± 24 | 0.8% | 38.5 | myopic | ECCE |
| 2 | Bhagwandien et al.[[18](#_ENREF_18)] | 2006 | 3,094 (4262) | 52.3 | 0.62% | 15.46 | mixed | ECCE, Phaco |
| 3 | Erie et al.[[24](#_ENREF_24)] | 2006 | 7,137 (10,256) | 240 total | 1.8% | not mentioned | mixed | ECCE, Phaco |
| 4 | Tuft (2006) et al.[[15](#_ENREF_15)] | 2006 | 45,520 (63298) | 17.5 | 0.41% | 9.8 | mixed | ECCE, Phaco |
| 5 | Alio et al.[[17](#_ENREF_17)] | 2007 | 274 (439) | 61.5 ± 29.6 | 2.7% | not mentioned | myopic | Phaco |
| 6 | Ripandelli et al.[[12](#_ENREF_12)] | 2007 | 453 (453) | 60 total | 3.1% | not mentioned | emmetropic | Phaco |
| 7 | Sheu (2007) et al.[[13](#_ENREF_13)] | 2007 | 9,388 | 54.99 ± 15.53 | 1.2% | 31.75±18.51 | mixed | ECCE, Phaco |
| 8 | Sheu (2010) et al.[[14](#_ENREF_14)] | 2010 | 9,388 | 79.21 ± 15.56 | 2.31% | 40.6±13.34 ECCE | mixed | Phaco |
| 9 | Clark (2012) et al.[[22](#_ENREF_22)] | 2012 | 65,055 | 79.2 | 0.7% | not mentioned | mixed | Phaco |
| 10 | Quek et al.[[11](#_ENREF_11)] | 2012 | 24,846 | 84 | 0.16% | 15.7 | mixed | ICCE, ECCE, Phaco |
| 11 | Clark (2011) et al.[[21](#_ENREF_21)] | 2011 | 129,982 | 79.2 | 0.68% | 11 | mixed | ECCE, Phaco |
| 12 | Hermann et al.[[26](#_ENREF_26)] | 2012 | (798) | 180 | 0.27% | 51.72 | mixed | not mentioned |
| 13 | Lin et al.[[10](#_ENREF_10)] | 2012 | 9,184 | 24.16±28.16 | 0.84% | 38.28±22.56 | mixed | ECCE, Phaco |
| 14 | Olsen et al.[[28](#_ENREF_28)] | 2012 | 7,856 (12,222) | 64.8 | 0.39% | 26.5 | mixed | Phaco |
| 15 | Tuft (2012) et al.[[4](#_ENREF_4)] | 2012 | 45,520 (63,298) | 44.8 | 1.17% | not mentioned | mixed | not mentioned |
| 16 | Al Muammar et al.[[16](#_ENREF_16)] | 2013 | 721 (high myopia 352; control 500) | 47.8 ±31.2 myopia 38.7 ± 18.2 controls | 2.8% myopia 0.4% controls | not mentioned | myopic and control | ECCE, Phaco |
| 17 | Bjerrum et al.[[19](#_ENREF_19)] | 2013 | 202,226 | 120 | 4.23% | not mentioned | mixed | Phaco |
| 18 | Daien et al.[[8](#_ENREF_8)] | 2015 | 1,787,021 (2,680,167) | 48 | 0.99% | 7.9 | mixed | ECCE, Phaco |
| 19 | Chen et al.[[20](#_ENREF_20)] | 2016 | 260 | not mentioned | not mentioned | not mentioned | not mentioned | not mentioned |
| 20 | Day et al.[[23](#_ENREF_23)] | 2016 | (61,907) | 2.4 | 0.21% | 44 days PCR 6.3 months without PCR | CS with PCR | Phaco |
| 21 | Laube et al.[[9](#_ENREF_9)] | 2017 | 7,886 (13,925) | 45 | 3.55% | 21.57 | mixed | Phaco |

CS= Cataract surgery, Phaco= Phacoemulsification, ECCE=extracapsular cataract extraction, PCR=posterior capsule rupture, ICCE= intracapsular cataract extraction,
